# Non-Sustained Ventricular Tachycardia as a Sign of Lung Cancer

**DOI:** 10.7759/cureus.6090

**Published:** 2019-11-07

**Authors:** Shujaul Haq, Sohaib Roomi, Bilal H Lashari, Muhammad Atique Alam Khan

**Affiliations:** 1 Internal Medicine, Abington Hospital - Jefferson Health, Abington, USA

**Keywords:** non-small cell lung cancers (nsclc), ventricular tachycardia, dysphagia, metastasis

## Abstract

The leading cause of death due to malignancy in the USA is lung cancers. They can be divided into small cell lung cancer and non-small cell lung cancer. Of the latter, adenocarcinoma comprises the majority of lung cancers. Manifestations of lung cancer can be divided into thoracic, extra-thoracic and paraneoplastic syndromes. We describe a case of ventricular tachycardia in a patient who presented with dysphagia, ultimately found to have a non-small cell lung cancer invading the esophagus and heart.

## Introduction

Lung and bronchial cancers are the leading cause of death in the United States and in the world [1-2]. According to the SEER cancer statistics review, in the USA, the estimated incidence of lung and bronchus cancers for the year 2018 was about 234,030, and estimated deaths were about 154,050. The overall 5-year survival rate was 18.6% [1].

Lung cancers can broadly be divided into two categories which comprise about 90% of all lung cancers, small cell lung cancer (SCLC) and non-small cell lung cancer (NSCLC) [3]. NSCLC comprises adenocarcinoma, which is the most common type, comprising about 40% of all lung cancers. Squamous cell carcinoma comprises about 25%-30% and large cell carcinoma comprises about 5%-10%. One of the biggest risk factors for the development of lung cancer is smoking [4], but it can also occur without any history of smoking. Other risk factors that have been studied that can cause lung cancer are second-hand smoking, radon gas, asbestos exposure, air pollution, personal and family history with an association of genes such as TP53 [5].

The manifestations of lung cancer can be divided into thoracic and extra-thoracic manifestations, and paraneoplastic syndromes. Thoracic manifestations can include a variety of signs and symptoms. It can cause a cough in more than 50% of patients with the production of sputum. The sputum can also be blood-streaked that represents hemoptysis [6]. Lung cancer can also involve the recurrent laryngeal nerve that can lead to hoarseness. Other local symptoms can include invasion of the esophagus leading to dysphagia and extension into the heart which can lead to pericardial effusions and arrhythmias [7].

We describe a case of ventricular tachycardia as a manifestation of lung cancer in a patient who initially presented with dysphagia but was diagnosed with NSCLC invading the esophagus and heart along with some displacement of the latter.

## Case presentation

Our patient is an 83-year-old female with a medical history of hypertension and a recent history of dysphagia who presented to the hospital with gradually worsening dysphagia, primarily to solids. The patient had a self-reported weight loss of about 70 pounds last year before her presentation to the hospital and had been undergoing investigations at another hospital for ruling out malignancies. Per patient, she was seen at the previous hospital about one month ago and an upper GI endoscopy was normal. Previously, the patient had radiographic findings suggestive of achalasia and esophageal manometry was planned. Other symptoms such as odynophagia, nausea, vomiting, change in bowel habits, melena, bright red blood per rectum were absent. Family history was noncontributory for any malignancies.

An initial chest x-ray was unrevealing. A complete metabolic panel revealed normal electrolytes and LFTs. A complete blood count showed anemia with hemoglobin of 11.4 and hematocrit of 33.3.

She was evaluated by GI and an esophageal manometry was performed. It showed evidence of achalasia (subtype II) and subsequently, an esophagogastroduodenoscopy (EGD) was performed with balloon dilation and Botox injections were successfully applied for sphincter relaxation. This did not decrease her symptoms of dysphagia and she was started on total parenteral nutrition for support.

**Figure 1:**
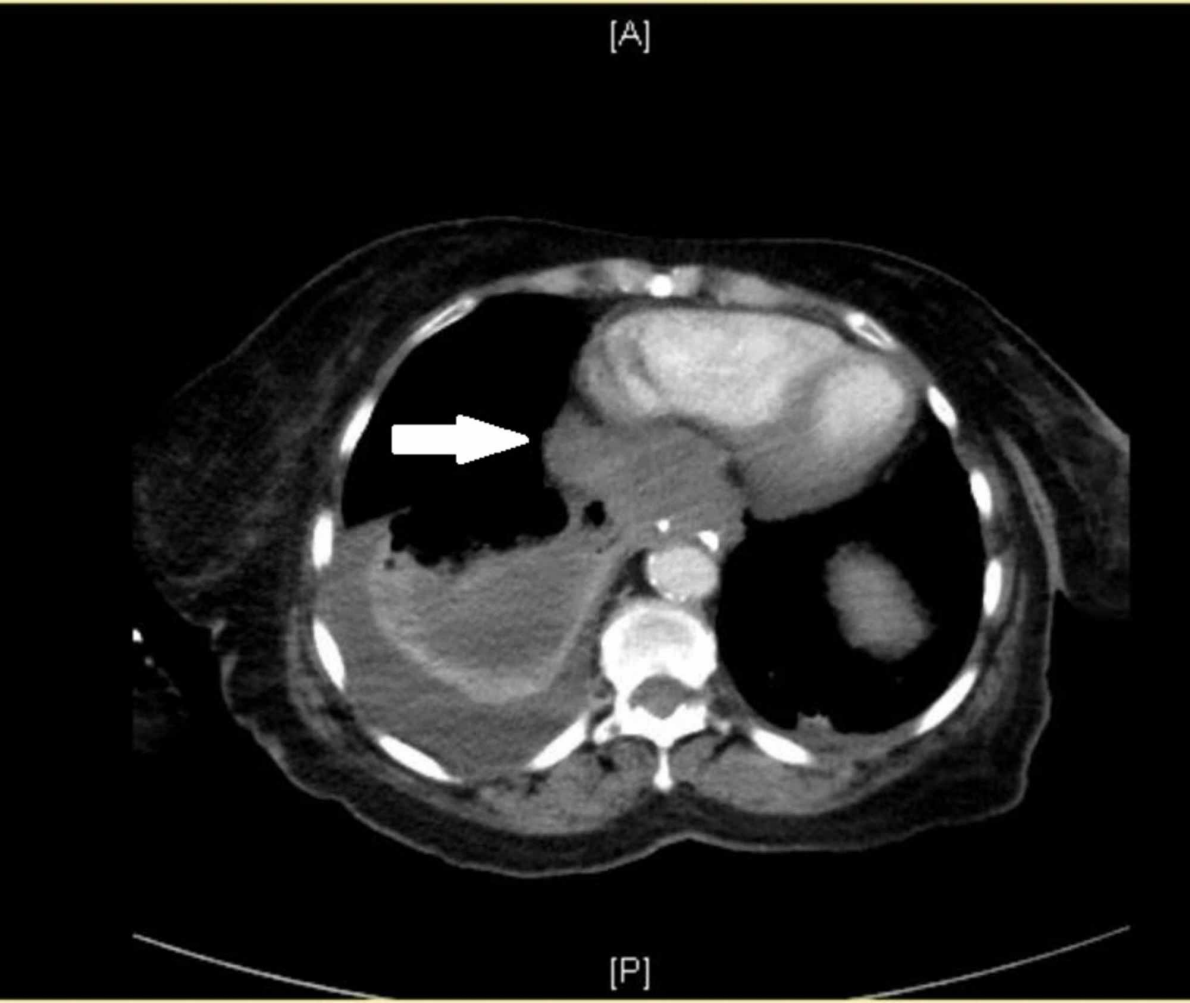
Arrow shows lung mass measuring about 40 mm x 75 mm. It also demonstrates the displacement of the heart.

A possibility of extrinsic compression needed to be ruled out and a computed tomography (CT) chest was performed. It revealed a soft tissue mass posterior to the heart slightly displacing it anteriorly and adjacent to the esophagus which might have been invading or arising from the esophagus. (Figure 1).

**Figure 2:**
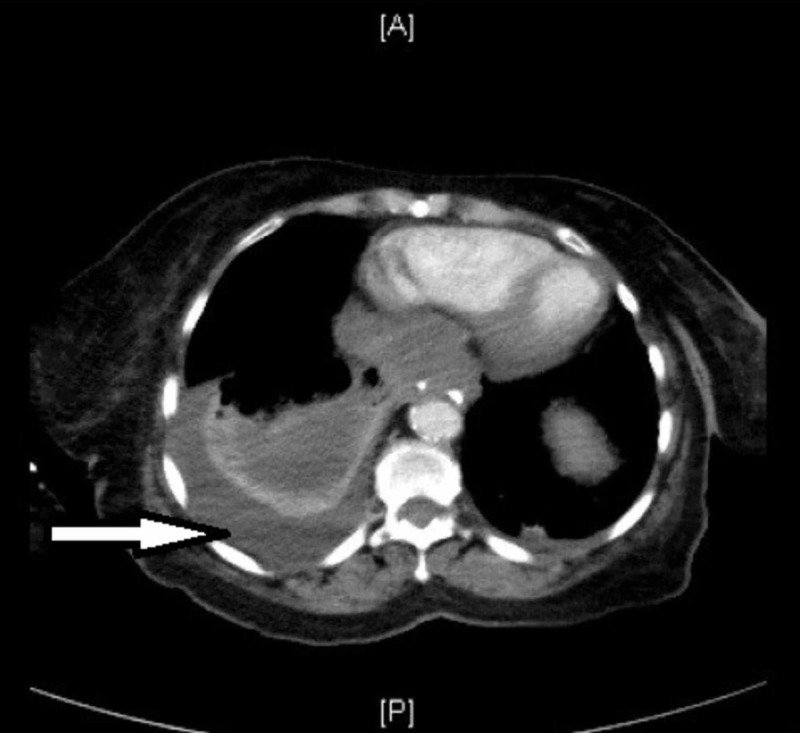
Arrow demonstrating right-sided pleural effusion.

**Figure 3:**
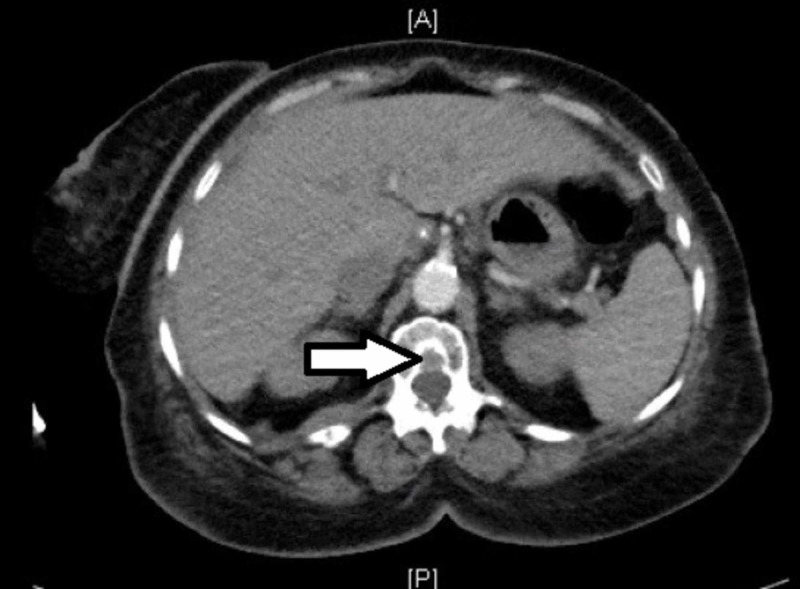
Arrow demonstrating vertebral metastasis on computed tomography scan.

It also revealed a partially loculated right moderate pleural effusion and enlarged calcified hilar lymph nodes. CT also demonstrated lucent lesions in the several thoracic lumbar vertebrae concerning for metastasis. (Figures 2 and 3).

Meanwhile, records acquired from the previous hospital demonstrated NSCLC on a lung biopsy and it was likely that it was the culprit behind the external compression. Surgery was consulted and an open gastric tube was placed for nutrition. Hematology and Oncology were consulted for the eventual treatment of the NSCLC.

The clinical course became complex when the patient had a development of new-onset atrial fibrillation and runs of non-sustained ventricular tachycardia. The patient, however, remained hemodynamically stable and did not complain of any chest pain or shortness of breath. An echocardiogram showed normal left ventricular (LV) function with an EF of 60%-65%. Mild mitral regurgitation and elevated right ventricular systolic pressure (RVSP) at 45-50 mm Hg. No pericardial effusion or any other abnormality was demonstrated. She was started on amiodarone and anticoagulated with Apixaban 2.5 mg twice daily. She continued to have runs of non-sustained ventricular tachycardia. It was adequately controlled by the administration of amiodarone. Meanwhile, the pathology was reviewed by Hematology and Oncology and it suggested NSCLC with squamous differentiation. She was diagnosed with stage 4 NSCLC with metastasis to the spine and ribs and an MRI of the head also demonstrated a possible area of metastasis in the brain.

After a discussion with the patient, it was decided that a palliative approach to treatment will be adapted with local radiotherapy in combination with systemic chemotherapy.

## Discussion

Primary cardiac tumors are very rare and according to a study comprise only up to 0.03% of cases with most of the tumors being benign. Metastasis to the heart is not uncommon and therefore in a lot of cases, a primary lung malignancy can metastasize to the heart and can cause atrial and ventricular arrhythmias which could be sustained or non-sustained. Our review of the literature shows cases where cardiac arrhythmias can be related to lung cancer [8-11].

Tumors can spread to the heart through different routes. There can be a direct extension of tumor cells, hematogenous spread, lymphatic system or diffusion through either the inferior vena cava or the pulmonary veins [12].

Tumor with the highest rate of metastasis to the heart is pleural mesothelioma, with squamous cell carcinoma of the lung causing about only 18% of cases. Mostly, about 70% of the time, cardiac metastases involved the pericardium, followed by the epicardium, myocardium, and endocardium in a decreasing fashion [12]. We can also see signs of valve disease or heart failure. If the pericardium is involved, the patient might present with tachycardia and tamponade physiology due to neoplastic effusions. If the myocardium is involved, we can see conduction defects and arrhythmias such as atrial fibrillation or ventricular tachycardia just like in our patient. Atrioventricular blocks are also seen that can be correlated with the neoplasm invading the conduction system. Some cardiac arrhythmias can also be related to systemic chemotherapy [13]. In our patient, no radiotherapy or chemotherapy had been administered when the arrhythmias occurred suggesting a link with the metastatic lung cancer.

Various imaging modalities can be used to assess cardiac metastasis. An echocardiogram (trans-thoracic and trans-esophageal) has a very good diagnostic accuracy to look for cardiac metastasis [14]. It can also reveal any malignant pericardial effusions and so it is usually a test of choice in the initial part of the workup. A CT scan can also provide a lot of information about any cardiac involvement as it has a larger field of vision and can see other surrounding structures and a higher resolution which aids in diagnosing effusions and masses. It also visualizes the lymph nodes and we can get a better sense of anatomy. A positron emission tomography scan shows focal uptake of tracer in areas of metastasis. This too can aid in our diagnosis of cardiac metastasis [15]. But by far the best test for assessing cardiac metastases is magnetic resonance imaging (MRI) of the heart. Its better tissue characterization, higher contrast resolution, and multi-planar cardiac imaging make it the gold standard for cardiac metastasis [15].

At the time of the diagnosis of metastatic cancer, the management is usually palliative rather than curative. In line with that, the management of cardiac metastasis usually involves dealing with primary cancer and if manifestations of cardiac involvement are seen such as described above, then additional therapeutic measures can be performed. For example, for a malignant tamponade, a percutaneous pericardiocentesis is necessary or if the conduction system is involved, a pacemaker can be implanted. Cardiac surgery is usually reserved for masses that are solitary and the patient has a good prognosis [16]. In our patient, the recurrent NSVTs were treated with Amiodarone a good control of the ventricular arrhythmia was seen. For our patient, it was planned that a palliative approach will be taken with radiation and chemotherapy but these therapies are also associated with myocardial fibrosis, pericarditis, and cardiac failure [16].

## Conclusions

Primary cardiac tumors are rare but cardiac metastases are commonly seen with various types of cancers. When suspected, they should be investigated thoroughly and managed according to the symptomatology that they present with.
